# MogaDepth: Multi-Order Feature Hierarchy Fusion for Lightweight Monocular Depth Estimation

**DOI:** 10.3390/s26020685

**Published:** 2026-01-20

**Authors:** Gengsheng Lin, Guangping Li

**Affiliations:** School of Information Engineering, Guangdong University of Technology, Guangzhou 510000, China; 2112303045@mail2.gdut.edu.cn

**Keywords:** monocular depth estimation, lightweight model, multi-order feature interactions, self-supervised, edge devices

## Abstract

Monocular depth estimation is a fundamental task with broad applications in autonomous driving and augmented reality. While recent lightweight methods achieve impressive performance, they often neglect the interaction of **mid-order semantic features**, which are crucial for capturing object structures and spatial relationships that directly impact depth accuracy. To address this limitation, we propose **MogaDepth**, a lightweight yet expressive architecture. It introduces a novel **Continuous Multi-Order Gated Aggregation (CMOGA)** module that explicitly enhances mid-level feature representations through multi-order receptive fields. In addition, we present **MambaSync**, a global–local interaction unit that enables efficient feature communication across different contexts. Extensive experiments demonstrate that MogaDepth achieves highly competitive or superior performance on KITTI, improving key error metrics while maintaining comparable model size. On the Make3D benchmark, it consistently outperforms existing methods, showing strong robustness to domain shifts and challenging scenarios such as low-texture regions. Moreover, MogaDepth achieves an improved trade-off between accuracy and efficiency, running up to 13% faster on edge devices without compromising performance. These results establish MogaDepth as an effective and efficient solution for real-world monocular depth estimation.

## 1. Introduction

Depth estimation plays a vital role in a wide range of applications, including autonomous driving, 3D reconstruction [1], augmented reality, robotics, and medical imaging. Accurate depth perception enables the understanding of spatial scene structures, object positioning, and metric distances, which are foundational for tasks such as semantic segmentation, autonomous navigation, robotic surgery assistance, and human pose estimation.

Traditional depth sensing systems, such as radars, LiDARs, or stereo cameras, often produce sparse depth representations and are constrained by cost, power consumption, and hardware complexity. Depth cameras using stereo disparity, structured light, or time-of-flight methods provide denser outputs, but they suffer from photometric sensitivity, limited range, and motion blur. These limitations hinder performance in dynamic environments and long-range scenes, prompting the development of monocular depth estimation methods.

Monocular depth estimation based on supervised learning, particularly when utilizing convolutional neural networks (CNNs), attains superior results when trained with high-quality annotated depth data [2,3,4]. However, the scarcity and high cost of dense annotations present practical implementation challenges. Consequently, self-supervised methods have emerged as a promising alternative by exploiting geometric constraints from stereo image pairs or monocular video sequences [5,6,7,8].

Self-supervised monocular depth estimation can be broadly categorized into two primary paradigms: **stereo-based approaches** [5] and **video-based frameworks** [6,8,9]. Stereo-based techniques estimate depth by analyzing geometric disparities between synchronized rectified image pairs. While these methods eliminate the need for explicit camera motion estimation, their dependency on dual-camera systems imposes stringent calibration requirements and synchronization constraints, thereby restricting their scalability in real-world deployments. By contrast, video-based methodologies leverage sequential frames captured by a monocular camera in motion, necessitating integration with pose estimation networks to infer camera motion trajectories [6,9,10,11]. Despite this added complexity, monocular video systems demonstrate enhanced deployment flexibility as they circumvent hardware limitations associated with stereo configurations, rendering them particularly advantageous for edge computing applications on resource-constrained mobile platforms or embedded systems.

Recent progress in video-based paradigms has addressed key challenges through multi-faceted optimization strategies: generative adversarial networks (GANs) [12] mitigate occlusion artifacts and dynamic scene complexities, semantic supervision modules [13,14] enhance depth consistency, and advanced loss formulations [6] improve prediction accuracy. Concurrently, hybrid architectures integrating convolutional neural networks (CNNs) with vision transformers (ViTs) [15] have attracted significant interest due to their complementary strengths—CNNs excel at hierarchical feature aggregation through spatially-localized receptive fields, while ViTs capture global dependencies via self-attention mechanisms. However, existing hybrid models still face unresolved bottlenecks that impede their practical deployment and performance.

Specifically, three core limitations persist in current state-of-the-art methods. First, in terms of computational complexity, ViTs exhibit quadratic computational complexity relative to input resolution, making them infeasible for high-resolution depth estimation in low-power, resource-constrained environments. Second, regarding feature representation, most lightweight depth estimation models (e.g., R-MSFM [11,16], Lite-Mono [10]) focus predominantly on low-order cues (e.g., edges and textures) or high-order semantics (e.g., global context), lacking explicit mechanisms to model mid-order features (e.g., object parts, contours, and intermediate spatial structures). This oversight leads to oversmoothed depth maps and compromised structural fidelity. Third, in terms of deployment adaptability, a critical trade-off exists between accuracy, computational efficiency, and deployment flexibility—existing lightweight models often sacrifice depth estimation accuracy to reduce complexity, failing to meet the requirements of real-time embedded applications.

To address these limitations, we propose a lightweight and efficient depth estimation model with a hybrid CNN and Mamba architecture, which constitutes an innovative amalgamation for enhancing the accuracy of depth prediction while maintaining computational efficiency. Specifically, inspired by EfficientViM [17], we design the **MambaSync** module to replace the computationally expensive self-attention mechanism with a structured state-space model (SSM). This SSM achieves linear computational complexity in both time and space, significantly reducing memory usage and enabling scalability to high-resolution feature maps. Unlike conventional attention mechanisms that process all token pairs indiscriminately, Mamba leverages a parameter-efficient and sequence-aware formulation that captures long-range dependencies with greater efficiency, while supporting parallel sequence processing and exhibiting strong inductive biases for spatial and temporal continuity—traits particularly advantageous for dense prediction tasks where both global coherence and local detail are critical. Complementing this, the **CMOGA** module enhances spatial and semantic representation through hierarchical fusion of low-, mid-, and high-order features, explicitly addressing the gap in mid-order feature modeling. Together, these two components enable **MogaDepth** to accomplish accurate and consistent depth predictions while maintaining computational efficiency suitable for real-time and resource-constrained applications.

Our main contributions can be summarized as follows:

- We propose a lightweight, end-to-end self-supervised depth estimation architecture that achieves strong performance while minimizing parameters and floating-point operations (FLOPs).
- Our model achieves highly competitive results on the KITTI benchmark [18] and generalizes well to the Make3D dataset [19], demonstrating strong cross-domain robustness.
- We validate MogaDepth’s efficiency and real-time performance on NVIDIA RTX 3090 and Jetson Xavier, demonstrating its practical deployability.

## 2. Related Work

Monocular depth estimation from a single image is inherently under-constrained due to perspective projection. Deep learning methods address this challenge by learning hierarchical multi-scale features, and current approaches can be broadly categorized into supervised and self-supervised paradigms. This section reviews relevant advances in these areas, with a specific focus on self-supervised methods (organized by their technical paradigms) and lightweight model designs, while analyzing existing limitations to motivate the contributions of this work.

### 2.1. Supervised Depth Estimation

Supervised monocular depth estimation methods train CNN-based models using paired RGB-depth data, which provides direct supervision for depth prediction. Early works [3] pioneered multi-scale prediction architectures to capture spatial details at different resolutions. Subsequent improvements integrated complementary cues (e.g., semantic segmentation, motion information) and advanced fusion strategies [20,21,22,23] to enhance depth consistency and accuracy. While supervised methods can achieve high performance when sufficient annotated data is available, their practical applicability is severely constrained by the scarcity and high annotation cost of large-scale dense depth datasets. This fundamental limitation has driven the shift toward self-supervised depth estimation, which leverages intrinsic geometric constraints of visual data to avoid reliance on manual annotations.

### 2.2. Self-Supervised Monocular Depth Estimation

Self-supervised methods eliminate the need for annotated depth data by exploiting photometric consistency across multiple views (stereo pairs or video sequences). Based on the source of visual cues, they can be divided into two primary paradigms that align with the classification in the Introduction: **stereo-based approaches** and **video-based frameworks**.

#### 2.2.1. Stereo-Based Approaches

Stereo-based self-supervised methods estimate depth by analyzing geometric disparities between synchronized and rectified left-right image pairs. Godard et al. [24] laid the foundation for this paradigm by introducing left-right consistency constraints to regularize depth prediction, effectively mitigating ambiguous depth estimates. These methods offer the advantage of avoiding explicit camera motion estimation, simplifying the model pipeline. However, their inherent dependency on dual-camera systems imposes stringent requirements on hardware calibration and temporal synchronization. Such constraints significantly limit their scalability in real-world deployments (e.g., on resource-constrained monocular devices), restricting their applicability in broader edge computing scenarios.

#### 2.2.2. Video-Based Frameworks

Video-based self-supervised methods leverage sequential frames captured by a moving monocular camera, requiring joint optimization of depth estimation and camera pose prediction networks to infer 3D scene structure. Monodepth2 [6] represents a landmark work in this area, introducing multi-scale loss, auto-masking, and minimum reprojection error strategies to address key challenges such as static scene ambiguity and occlusion artifacts. Zhou et al. [8] further advanced the field by proposing a dual-branch encoder-decoder architecture for end-to-end joint depth and pose estimation, enabling unsupervised learning purely through photometric loss. Subsequent works have focused on refining network components and fusion strategies to enhance robustness and accuracy. For example, SPIdepth [25] emphasizes the design of more robust pose estimation networks to improve cross-scene generalization, while ProDepth [26] introduces probabilistic multi-frame fusion techniques to handle dynamic scenes more effectively. Despite these advances, video-based frameworks still face critical limitations: existing models either rely on computationally expensive architectures (e.g., large CNNs or ViTs) that hinder real-time deployment, or sacrifice depth accuracy when pursuing lightweight designs—especially in scenarios requiring fine-grained structural detail.

### 2.3. Lightweight Depth Estimation Models

With the growing demand for deploying depth estimation models on resource-constrained devices (e.g., mobile phones, embedded systems), lightweight depth estimation has emerged as a key research focus. The core goal of this area is to balance high depth estimation accuracy with reduced model size and computational cost. Current lightweight design strategies can be broadly categorized into three types: network pruning [27], knowledge distillation [28], and efficient architectural design [29,30,31]. Network pruning removes redundant connections and neurons to compress model size, while quantization reduces memory usage by adopting lower-bit representations. Knowledge distillation transfers learned features from large, high-performance “teacher” models to compact “student” models, preserving performance with reduced complexity. Among these strategies, efficient architectural design is the most actively explored direction, which leverages techniques such as depthwise separable convolutions, residual connections, and lightweight attention mechanisms, often combined with multi-scale feature fusion to retain spatial detail and semantic consistency.

Representative lightweight frameworks include R-MSFM [12,16], which employs fixed-resolution multi-layer depth optimization and multi-scale feature fusion to improve accuracy. However, its fixed-resolution design limits multi-scale perception capabilities and fails to fully exploit mid-order features (e.g., object contours and part structures), leading to suboptimal structural fidelity in depth predictions. Hybrid CNN-Transformer architectures have also been explored as alternatives to pure CNN-based lightweight models. For instance, Lite-Mono [10] combines the strengths of CNNs in local feature extraction with the global context modeling capability of Transformers. While modules such as CDC (continuous dilated convolution) and LGFI (lightweight global feature interaction) enhance multi-scale feature fusion, these designs inherently focus on integrating low-order cues (edges, textures) or high-order semantics (global context) without explicit mechanisms to model mid-order feature interactions. This oversight results in oversmoothed depth maps and compromised structural detail—gaps that motivate the design of our proposed model.

To address the aforementioned limitations in mid-order feature modeling and computational efficiency, this work proposes the **Continuous Multi-Order Gated Aggregation (CMOGA)** module, which explicitly captures interactions between low-, mid-, and high-order features to bridge local detail and global context. Complementing this, the **MambaSync** module leverages a structured state-space model (SSM) to achieve linear computational complexity in long-range dependency modeling, avoiding the quadratic complexity of traditional Transformers. The synergistic integration of these two modules enables our MogaDepth model to achieve both lightweight deployment and high structural fidelity in depth predictions.

## 3. Methodology

Although Lite-Mono [10] provides a strong lightweight baseline by combining CNNs and Transformers, its encoder still faces challenges in preserving fine-grained details and achieving efficient global modeling. To address these issues, we mainly focus on improving the encoder. Specifically, we replace the CDC module with the proposed CMOGA, which enhances mid-order and boundary-aware representations, and substitute the LGFI module with MambaSync, a scalable state-space model that efficiently captures long-range dependencies. These modifications build on the strengths of Lite-Mono while further improving detail preservation and computational efficiency.

**Figure 1:**
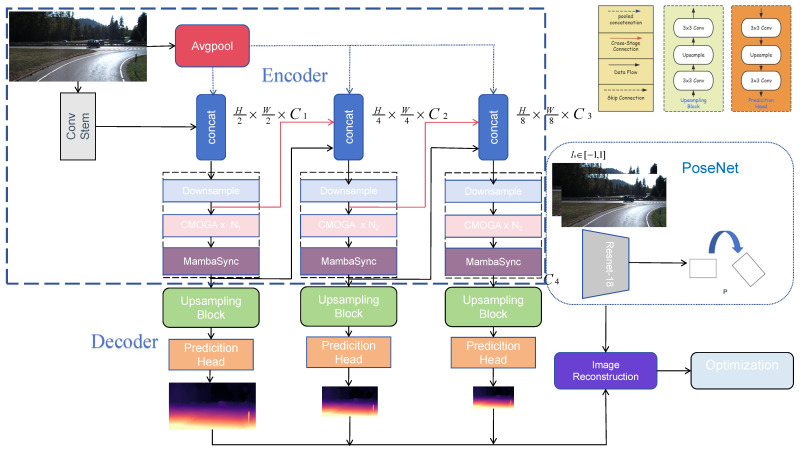
Overview of the proposed MogaDepth. MogaDepth has an encoder-decoder DepthNet for depth prediction, and a commonly used PoseNet [2,32] to estimate poses between adjacent monocular frames. The encoder of the DepthNet consists of four stages, and it uses CMOGA modules and MambaSync modules to extract rich hierarchical features.

The overall architecture of the proposed MogaDepth is presented in Figure 1.

### 3.1. MogaDepth Encoder

As shown in the blue box of Figure 1, the proposed MogaDepth Encoder adopts a four-level multi-scale feature aggregation architecture, which is theoretically motivated by the inherent demand of dense monocular depth estimation for both fine-grained spatial details and high-level semantic context. This architecture realizes efficient representation learning through progressive downsampling and cross-stage feature fusion, striking a balance between feature richness and computational efficiency for resource-constrained scenarios. An input RGB image with dimensions H×W×3 first passes through an initial convolutional backbone composed of two groups of 3×3 convolutional layers (stride = 1). This backbone is designed to suppress high-frequency noise while extracting primary edge and texture features, generating the first-stage feature map with dimensions $\frac{H}{2}\times \frac{W}{2}\times C_{1}$. For the second stage, to mitigate spatial information loss caused by direct downsampling, we concatenate the first-stage features with resolution-matched input images (obtained via average pooling)—a strategy inspired by ESPNetv2 [33] that is proven to preserve low-level spatial cues critical for depth boundary prediction. The concatenated feature map is then downsampled to $\frac{H}{4}\times \frac{W}{4}\times C_{2}$ via a 3×3 convolution with stride = 2.

Cross-stage feature correlations are established by cascading features from previous downsampling layers (similar to ResNet’s residual connections), which theoretically ensures unobstructed gradient flow during deep network training and avoids the vanishing gradient problem. For subsequent third and fourth stages, we integrate CMOGA modules and MambaSync modules in a cascaded manner—this integration is not arbitrary but a targeted solution to two core limitations of existing CNN-based encoders: (1) insufficient modeling of mid-order feature interactions (e.g., object contours and part structures) that bridge low-level details and high-level semantics; (2) inefficient global context modeling with prohibitive computational complexity. Following the same downsampling principle, the encoder generates high-order semantic features with dimensions $\frac{H}{8}\times \frac{W}{8}\times C_{3}$ and $\frac{H}{16}\times \frac{W}{16}\times C_{4}$, ultimately forming a multi-scale feature pyramid that balances fine-grained information preservation and deep semantic extraction.

**Figure 2:**
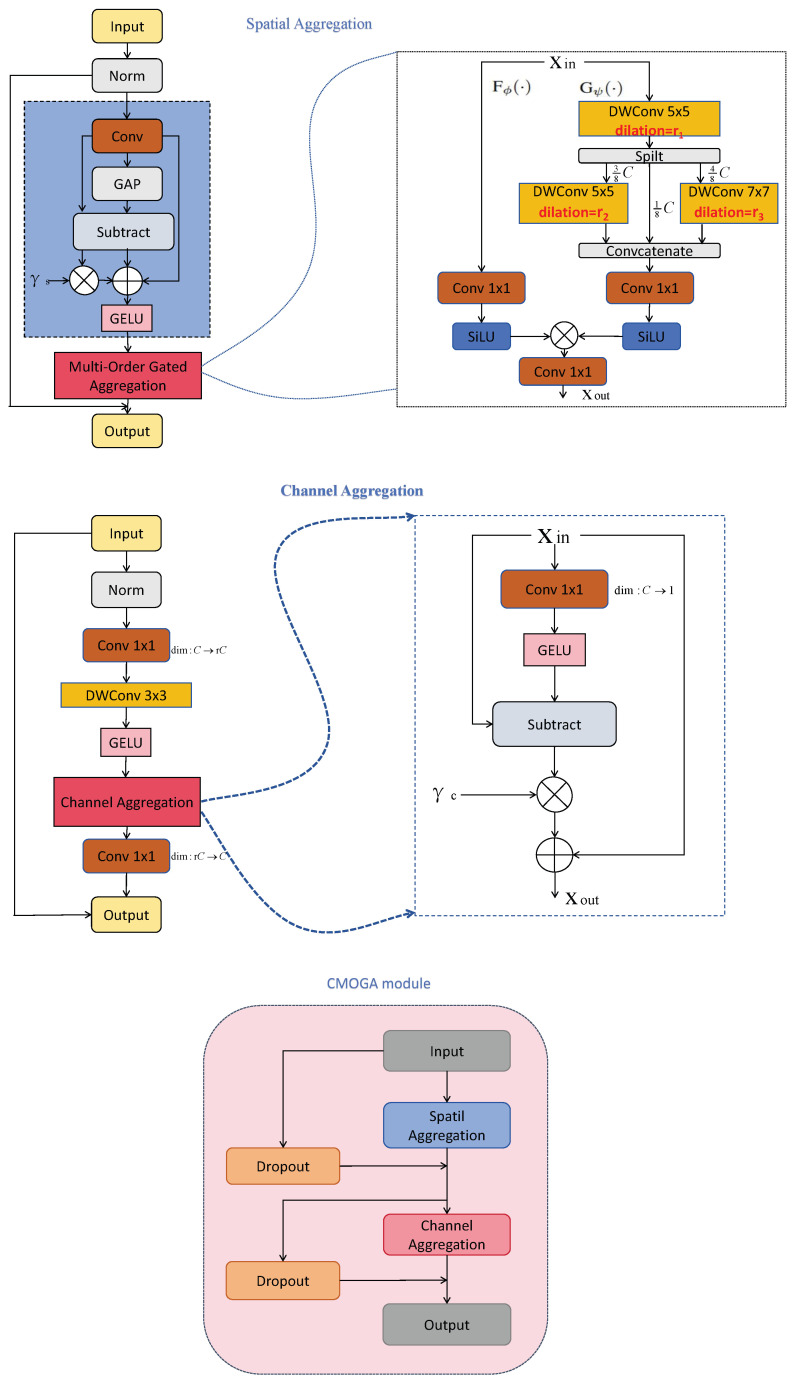
Structure of the proposed CMOGA module. The diagram explicitly presents the sequential data flow of the CMOGA module: the input feature map first enters the SA branch, which captures multi-scale spatial context via depthwise convolutions with variable dilation rates ($r_{1},r_{2},r_{3}$). The SA output is then transmitted to the CA branch for modeling inter-channel semantic dependencies through attention mechanisms. Two Dropout layers (connected to the SA and CA stages respectively) serve as auxiliary components to prevent overfitting and improve generalization. In each network stage, the CMOGA module (configured with distinct dilation rate combinations) is repeated $N_{i}$ times to deepen feature abstraction and strengthen mid-order feature interaction.

Three distinct depth encoder variants have been systematically designed via modular architecture differentiation, with each variant featuring unique channel configurations and CMOGA block parameterization strategies. The technical specifications of these design paradigms are comprehensively documented in Table 1. Meanwhile, the architectural frameworks for the multi-order aggregation mechanism in the CMOGA module and the details of the MambaSync modules are visually analyzed in Figure 2.

#### 3.1.1. Continuous Multi-Order Gated Aggregation (CMOGA)

The CMOGA module is theoretically designed to address the mid-order feature modeling gap in existing lightweight monocular depth estimation models. Prior works predominantly focus on fusing low-order texture features or high-order semantic features, while ignoring mid-order features (e.g., object contours, part relationships) that are essential for accurate depth structure prediction. Conceptually inspired by MogaNet [34], the CMOGA module retains the core design of complementary *spatial aggregation (SA)* and *channel aggregation (CA)* branches, but tailors hyperparameters (dilation rates, number of repeated blocks) specifically for dense prediction tasks. This targeted adjustment effectively strengthens mid-order feature interaction without altering the fundamental architecture, achieving a balance between performance improvement and computational cost control.

##### Overall Architecture of CMOGA Module

The CMOGA module follows a residual learning framework to enhance multi-order feature interaction for dense depth prediction, which is proven to facilitate feature fusion by preserving original input information while adding enhanced representations. Given an input feature tensor $X\in R^{H\times W\times C}$ (where *H*, *W*, and *C* denote the height, width, and channel number of the feature map, respectively), the module generates the enhanced feature tensor $X_{\mathrm{CMOGA}}$ through sequential computation, formally defined as:

(1) $$X_{\mathrm{SA}}=F_{\mathrm{SA}}\left(X\right),$$

(2) $$X_{\mathrm{CMOGA}}=F_{\mathrm{CA}}\left(X_{\mathrm{SA}}\right)+X,$$

where $F_{\mathrm{SA}}\left(\cdot \right)$ and $F_{\mathrm{CA}}\left(\cdot \right)$ encapsulate the core operations of the Spatial Aggregation (SA) and Channel Aggregation (CA) branches (detailed in subsequent paragraphs). Notably, the residual connection +X ensures the preservation of original fine-grained features while fusing multi-order semantic representations, which is crucial for retaining depth boundary accuracy in dense estimation tasks.

##### Spatial Aggregation (SA) Branch

The SA branch captures multi-scale spatial context through a Feature Decomposition (FD) module followed by a Multi-Order Gated Aggregation (MOGA) block, a design that aligns with the human visual system’s hierarchical perception of spatial structures. The FD module suppresses redundant activations and highlights discriminative local features by modeling deviations between local responses and global averages, formulated as:

(3) $$\begin{array}{cc} Y & ={\mathrm{Conv}}_{1\times 1}\left(X\right), \end{array}$$

(4) $$\begin{array}{cc} Z & =\mathrm{GELU}\;\left(Y+\gamma_{s}⊙\left(Y-\mathrm{GAP}\;\left(Y\right)\right)\right). \end{array}$$

where $\gamma_{s}\in R^{C}$ is a learnable channel-wise scale parameter, and GAP(·) denotes global average pooling.

The output Z is further processed by parallel depthwise convolutions with dilation rates r=1,2,3—this multi-dilation design enables the branch to capture local textures (r=1), mid-range contours (r=2), and large-scale object structures (r=3) without increasing computational complexity. These multi-scale features are concatenated along the channel dimension and modulated with a SiLU-based gating mechanism to adaptively weight informative mid-order features:

(5) $$Y_{C}=\mathrm{SiLU}\left(\mathrm{Concat}\left(Y_{l},Y_{m},Y_{h}\right)\right),$$

where $Y_{l},Y_{m},Y_{h}\in R^{H\times W\times C/3}$ correspond to outputs from dilation branches with r=1,2,3 (channel number is split equally to match the concatenation operation). By tuning the dilation rates and block repetition times, this branch effectively reinforces mid-level spatial feature interactions that are critical for dense depth prediction.

##### Channel Aggregation (CA) Branch

The CA branch complements the SA branch by modeling inter-channel semantic dependencies, adopting a lightweight design to avoid excessive computational overhead. It consists of a sequence of normalization, point-wise, and depthwise convolutions, followed by a channel interaction mechanism, formulated as:

(6) $$Y=\mathrm{GELU}\;\left({\mathrm{DWConv}}_{3\times 3}\;\left({\mathrm{Conv}}_{1\times 1}\;\left(\mathrm{BatchNorm}2d\;\left(X\right)\right)\right)\right),$$

(7) $$Z={\mathrm{Conv}}_{1\times 1}\;\left(\mathrm{CA}\;\left(Y\right)\right)+X.$$

The channel interaction operation is defined as:

(8) $$\mathrm{CA}\;\left(X\right)=X+\gamma_{c}⊙\left(X-\mathrm{GELU}\;\left(XW_{r}\right)\right).$$

where $\gamma_{c}\in R^{C}$ is a learnable channel-wise adjustment parameter, and $W_{r}\in R^{C\times C/r}$ denotes a channel reduction mapping (with r=4 as the channel compression ratio in our experiments). The careful selection of hyperparameters ensures effective aggregation of mid-level semantic channels, improving cross-scale feature integration while maintaining lightweight properties.

While CMOGA leverages MogaNet’s foundational structure, its core innovation lies in the tailored modifications of hyperparameters and repetition strategies. These changes are specifically aimed at boosting mid-level feature interaction and multi-scale fusion—vital for single-image depth prediction. By enhancing feature representation density without compromising computational efficiency, our model achieves superior performance. The effectiveness of these hyperparameter settings is quantitatively validated through ablation studies in Secion 4.5.2, confirming their ability to improve multi-order feature fusion with negligible computational overhead increase. 

#### 3.1.2. MambaSync Module

**Figure 3:**
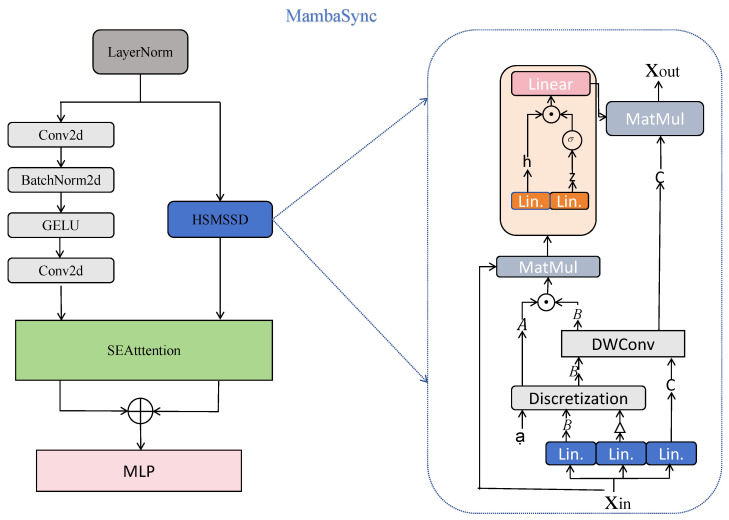
Structure of the proposed MambaSync module.

This module is theoretically designed to resolve the computational complexity bottleneck of global context modeling in existing Transformer-based depth estimation models. As shown in Figure 3, it employs two complementary branches to jointly capture local and global representations: a depthwise convolution (DWConv) branch for extracting fine-grained spatial features, and a **Hidden State Mixer based State Space Duality (HSMSSD)** [17] branch for hierarchical global context aggregation. To enhance feature fusion, a squeeze-and-excitation (SE) block is integrated to dynamically recalibrate channel-wise responses, facilitating adaptive balancing between local and global semantic cues.

The overall mechanism of MambaSync follows a dual-branch fusion framework, formally formulated as:

(9) $$F_{\mathrm{out}}=\mathrm{Mlp}\left(\mathrm{SE}\left(F_{\mathrm{local}}\right)+\mathrm{SE}\left(F_{\mathrm{global}}\right)\right),$$

where $F_{\mathrm{local}}\in R^{C\times H\times W}$ and $F_{\mathrm{global}}\in R^{C\times H\times W}$ are the output feature tensors from the DWConv and HSMSSD branches, respectively (*C*, *H*, *W* denote channel number, height, and width of feature maps). The SE block adaptively fuses local and global features by applying dynamic channel-wise weighting, ensuring that informative features are emphasized in the final representation.

##### Global Feature Extraction

Given an input feature tensor $X\in R^{C\times H\times W}$, the HSMSSD branch models long-range dependencies using a state space representation, which reduces computational cost from $O(LD^{2})$ to $O(ND^{2})$ (where L = H × W denotes the number of spatial tokens, D=C denotes feature dimension, and *N* denotes the number of hidden states). This linear complexity is achieved by projecting high-dimensional spatial features into a compressed latent space, enabling efficient global context modeling.

The input is first projected and convolved to generate latent state parameters:

(10) $$B,C_{\mathrm{proj}},\Delta t=\mathrm{Split}\left(\mathrm{DWConv}\left(\mathrm{Conv}1D\left(X\right)\right)\right),$$

where $B\in R^{C\times N}$ is the input mixing matrix, $C_{\mathrm{proj}}\in R^{N\times C}$ is the output projection matrix, and $\Delta t\in R^{N}$ is the learnable time-step vector for dynamic state transition. A normalized transition matrix A is then computed as:

(11) $$A=\mathrm{Softmax}\left(\Delta t+A_{\mathrm{init}}\right)\in R^{N\times L},$$

where $A_{\mathrm{init}}\in R^{N\times L}$ is the initial transition matrix initialized with small random values.

State update and output generation are performed as follows:

(12) $$h=x\cdot \left(A⊙B^{T}\right)\in R^{B_{\mathrm{batch}}\times C\times N},$$

(13) $$y=h\cdot C_{\mathrm{proj}}\in R^{B_{\mathrm{batch}}\times C\times L},$$

where $B_{\mathrm{batch}}$ denotes the batch size, $x\in R^{B_{\mathrm{batch}}\times C\times L}$ is the flattened projection of input X, h denotes the hidden state tensor, and y is the intermediate global feature tensor (resized back to $R^{B_{\mathrm{batch}}\times C\times H\times W}$ for subsequent fusion).

To enhance non-linearity and improve feature representation capability, a gated activation mechanism is introduced:

(14) $$h,z=\mathrm{Split}\left(\mathrm{Conv}1D\left(h\right)\right)\in R^{B_{\mathrm{batch}}\times D_{\mathrm{inner}}\times N},$$

(15) $$h=\mathrm{Conv}1D\left(h\cdot \mathrm{SiLU}\left(z\right)+h\cdot D\right)\in R^{B_{\mathrm{batch}}\times C\times N},$$

where $D_{\mathrm{inner}}=ssd_{\mathrm{expand}}\times C$ represents the dimension of the inner gated state, $ssd_{\mathrm{expand}}$ is a predefined expansion factor (set to 4 in our experiments), and $D\in R^{C\times N}$ is a learnable gating weight matrix.

##### Local Feature Extraction

To preserve spatial detail and texture information (critical for depth boundary prediction), the local branch consists of a lightweight convolutional block. Specifically, a 3×3 depthwise convolution is followed by batch normalization, GELU activation, and a 1×1 pointwise convolution to aggregate channel-wise responses while maintaining spatial resolution:

(16) $$F_{\mathrm{local}}={\mathrm{Conv}}_{1\times 1}\left(\mathrm{GELU}\left(\mathrm{BatchNorm}2d\left({\mathrm{DWConv}}_{3\times 3}\left(X\right)\right)\right)\right),$$

where $X\in R^{C\times H\times W}$ is the input feature tensor, BatchNorm2d denotes 2D batch normalization (explicitly specified to avoid ambiguity), and $F_{\mathrm{local}}\in R^{C\times H\times W}$ is the output local feature tensor.

The channel-wise recalibrated fusion of these two branches endows MambaSync with both robust modeling capability and high computational efficiency. Furthermore, the coordinated interaction between CMOGA (specialized in mid-order feature enhancement) and MambaSync (focused on efficient global context modeling) strikes a favorable balance between effective feature extraction and inference speed, forming a coherent system well-suited for resource-constrained dense prediction tasks such as monocular depth estimation.

### 3.2. Depth Decoder

Our decoder design follows Lite-Mono [10]. It progressively upsamples encoder features using bilinear interpolation combined with lightweight convolutional layers, while incorporating skip connections from three intermediate encoder stages. To enable multi-scale supervision, three prediction heads generate inverse depth maps at full, half, and quarter resolutions. This design achieves a favorable balance between accuracy and efficiency, ensuring the decoder remains lightweight and directly comparable across methods.

### 3.3. PoseNet

For relative pose estimation, we also adopt the design of LiteMono [10] to ensure a fair comparison. A ResNet18 backbone extracts features from image pairs, and a lightweight convolutional decoder estimates the 6-DoF camera transformation between consecutive frames. Since previous studies [13,35] report only marginal gains from more complex pose networks, we retain this efficient design in order to isolate and evaluate the contributions of our encoder.

### 3.4. Self-Supervised Learning

Following Lite-Mono [11], we adopt a self-supervised learning strategy based on image reconstruction, incorporating photometric consistency, edge-aware smoothness, and multi-scale supervision to train depth and pose networks without ground-truth labels.

**Dual-Network Joint Modeling** To decouple scene geometry from camera motion, we adopt a dual-network architecture:

DepthNet:

A convolutional encoder-decoder network that predicts inverse depth maps $d^{*}=\frac{1}{d}$ from a single target frame $I_{t}$. A sigmoid activation followed by linear scaling is used to constrain the depth range. Multi-scale outputs help capture both global structure and fine-grained details.

PoseNet:

A lightweight CNN that estimates the 6-DoF relative camera pose $T_{t\to s}\in SE\left(3\right)$ from adjacent frame pairs ($\left[I_{t-1},I_{t}\right]$ or $\left[I_{t},I_{t+1}\right]$), decomposed into rotation *R* and translation *t*.

**Differentiable View Synthesis** Given the predicted depth and pose, the target frame is reconstructed by warping source images using a differentiable projection model. Let $K\in R^{3\times 3}$ denote the camera intrinsic matrix (calibrated using KITTI’s average focal length as detailed in Section 4.1.1), $p_{s}\in R^{2}$ and $p_{t}\in R^{2}$ denote pixel coordinates in the source and target frames, respectively, and $D_{s}\left(p_{s}\right)$, $D_{t}$ denote the depth tensors at the corresponding pixels. The projection relation is formulated as:

(17) $$p_{t}=KT_{t\to s}D_{s}\left(p_{s}\right)K^{-1}p_{s},$$

(18) $${\hat{I}}_{t}=F\left(I_{s},D_{t},T_{t\to s},K\right),$$

**Photometric Reconstruction Loss** To guide learning, we employ a photometric loss that combines structural similarity (SSIM) and L1 pixel-wise error. Let α=0.85 denote the balance coefficient between the two terms:

(19) $$L_{p}\left({\hat{I}}_{t},I_{t}\right)=\alpha \cdot \frac{1-\mathrm{SSIM}({\hat{I}}_{t},I_{t})}{2}+\left(1-\alpha \right)\cdot ∥{\hat{I}}_{t}-I_{t}∥,$$

(20) $$L_{\min}=\min_{I_{s}\in \left[-1,1\right]}L_{p}\left({\hat{I}}_{t},I_{t}\right),$$

(21) $$M_{\mathrm{auto}}=\min_{I_{s}\in \left[-1,1\right]}L_{p}\left(I_{s},I_{t}\right)>\min_{I_{s}\in \left[-1,1\right]}L_{p}\left({\hat{I}}_{t},I_{t}\right),$$

where $M_{\mathrm{auto}}\in \left\{0,1\right\}$ is an auto-masking flag that filters dynamic objects or occluded regions (set to 1 if the source frame reconstruction error is larger than the target frame self-reconstruction error, and 0 otherwise).

**Edge-Aware Depth Smoothness** To encourage smooth depth predictions while preserving object boundaries, we incorporate an edge-aware regularization term. Let Ω denote the set of all pixel coordinates in the image:

(22) $$L_{\mathrm{smooth}}=\sum_{(x,y)\in \Omega }\left(|\partial_{x}d^{*}|\;\cdot \;e^{-|\partial_{x}I|}+\left|\partial_{y}d^{*}\right|\cdot e^{-|\partial_{y}I|}\right),$$

**Multi-Scale Supervision** Depth maps are predicted at three resolutions: full (H×W), half ($\frac{H}{2}\times \frac{W}{2}$), and quarter ($\frac{H}{4}\times \frac{W}{4}$). The overall training loss is computed as, where λ=0.1 is the weight of the smoothness term:

(23) $$L_{\mathrm{total}}=\frac{1}{3}\sum_{s\in \{1,\frac{1}{2},\frac{1}{4}\}}\left(L_{r}+\lambda \cdot L_{\mathrm{smooth}}\right),$$

## 4. Experiments

This chapter introduces the datasets used in training and validation, followed by comparative analyses demonstrating the advantages of the proposed method over existing approaches. Subsequent experiments evaluate the efficiency of inference, whereas ablation studies systematically verify the contributions of key components.

### 4.1. Datasets

#### 4.1.1. KITTI

The KITTI dataset [18], a widely recognized benchmark in autonomous driving and robotics research, comprises 61 meticulously curated stereo road scenes. Its multisensor data acquisition system integrates advanced instrumentation, including binocular camera arrays, 3D LiDAR units, and high-precision GPU/IMU inertial navigation modules. To facilitate algorithm development and performance evaluation, this study uses an eigenvalue-based hierarchical data partitioning strategy [32], structuring the original dataset into three distinct subsets: a training set containing 39,180 monocular image triplets with various traffic scenarios and illumination conditions, a validation set comprising 4424 samples, and a testing set consisting of 697 instances. This rigorous pyramidal data partition establishes a systematic framework for reliable performance validation.

Our self-supervised training methodology relies on the known intrinsic parameters of the camera, as originally described in [6]. By calculating the average focal length for all KITTI images, we implement a unified intrinsic calibration protocol during network optimization. During evaluation, predicted depth values are constrained within the [0, 80] m interval, following established industry standards for depth estimation benchmarks.

#### 4.1.2. Make3D

The Make3D dataset [19], a comprehensive benchmark for computer vision and depth estimation tasks, consists of 534 training images (2272 × 1704 pixels) and 134 test images. This data set uses multiview stereo image pairs (baseline distance 12 cm) captured through LiDAR camera fusion systems to generate geometrically diverse 3D scene representations. High-precision pixel-accurate depth annotations (error < 5%) are produced using photometric stereo reconstruction with rigid multi-sensor calibration. The dataset encompasses urban street environments, highway scenarios, and synthetic test cases with extreme lighting conditions, nonrigid occlusions, and physically implausible object configurations. These artificial augmentations aim to transcend the physical limitations of real-world data by creating photorealistic but structurally novel depth maps. Our experiments utilize Make3D to evaluate cross-domain generalization capabilities of models trained in KITTI, ensuring robust performance across heterogeneous outdoor environments with diverse visual characteristics and geometric complexities.

### 4.2. Implementation Details

Our method is implemented in PyTorch 2.6.0 and trained on a single NVIDIA RTX 3090 GPU using a batch size of 12. We adopt AdamW [36] as the optimizer with a weight decay of $1\times 10^{-2}$. To improve model generalization, we employ two complementary regularization strategies: DropPath regularization is applied within both the CMOGA and MambaSync modules, while data augmentation is adopted as a preprocessing step to enhance training robustness. Specifically, during training we perform the following augmentations with 50% probability each: horizontal flips, brightness adjustment (±0.2), saturation adjustment (±0.2), contrast adjustment (±0.2), and hue jitter (±0.1).

For models trained from scratch, the initial learning rate is set to $5\times 10^{-4}$, following a cosine annealing schedule [37], and the total number of training epochs is set to 30. Pretraining on ImageNet [38] significantly accelerates network convergence, so when pretrained weights are used, we reduce the initial learning rate to $1\times 10^{-4}$ and train for 30 epochs.

### 4.3. Evaluation Metrics

Depth estimation performance is evaluated using seven standard metrics from [2], capturing both absolute and relative errors while balancing global and local accuracy. AbsRel measures the percentage deviation from ground truth, and SqRel emphasizes larger errors via squared normalization. RMSE and RMSE log reflect global error, with the latter offering robustness to scale variation. The threshold metrics δ < 1.25, δ < 1.25^2^, and δ < 1.25^3^ indicate the percentage of pixels where estimates fall within 12.5%, 31.6%, and 70.8% of the true depth. Together, these metrics offer a comprehensive and practical evaluation of depth estimation models.

### 4.4. Results

#### 4.4.1. KITTI Results

Table 2 compares **MogaDepth** with existing lightweight depth estimation methods under strictly comparable parameter budgets (2.0–3.4 M), providing a comprehensive assessment of depth estimation accuracy across core metrics (Abs Rel, Sq Rel, RMSE, etc.). It should be emphasized that performance evaluation is based on peer-to-peer comparison of models with similar complexity—direct comparison with models of significantly higher parameter counts (e.g., DNA-Depth-B1 with 6.7 M parameters, Sun et al. with 10.9 M parameters) is inappropriate for lightweight model assessment. Objectively, MogaDepth does not achieve leading performance in all indicators among models with comparable parameter scales; instead, it demonstrates targeted optimization in key metrics while maintaining competitive performance in others, which aligns with the design goal of prioritizing inference efficiency for resource-constrained platforms.

Specifically, focusing on models with similar parameter sizes (2.0–3.4 M), our standard MogaDepth (3.4 M parameters) outperforms the representative lightweight architecture **LDA-Mono-L** (2.0 M parameters) in two core metrics sensitive to large-scale depth prediction errors and global consistency: the **SqRel metric is reduced from 0.765 to 0.745 (2.6% reduction)** and the **RMSE metric is reduced from 4.535 to 4.504 (0.68% reduction)**. From a theoretical perspective, the reduction in SqRel stems directly from the **CMOGA module’s mid-order feature modeling capability**: by explicitly capturing object contours and part-level spatial structures, CMOGA mitigates over-smoothing in texture-less regions and erroneous depth assignments at object boundaries—issues that often lead to large deviation errors heavily penalized by squared relative error calculations. The decrease in RMSE further validates the effectiveness of the **MambaSync module’s linear-complexity global context modeling**: unlike traditional Transformer-based attention mechanisms with quadratic complexity, MambaSync captures long-range spatial dependencies (e.g., relative depth between distant objects and background) without expanding parameter overhead, reducing the overall variance of depth predictions across the entire image and lowering the root mean square error. For other metrics, MogaDepth maintains performance comparable to state-of-the-art lightweight models: its Abs Rel (0.104) is on par with LDA-Mono-L (0.104), and the threshold-based accuracy metrics ($\delta <1.25^{2}$, $\delta <1.25^{3}$) reach 0.964 and 0.984 respectively—consistent with Lite-Mono (3.1M parameters, 0.963/0.983) and LDA-Mono-L (0.964/0.983), indicating that the efficiency-oriented architectural design does not lead to significant accuracy degradation.

**Figure 4:**
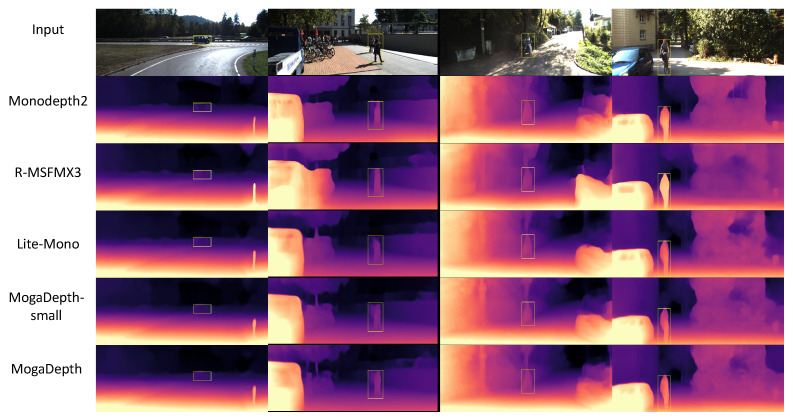
Qualitative results on KITTI. The depth maps below are predicted by Monodepth2 [6], R-MSFM3 [16], Lite-Mono [10], MogaDepth-small, and MogaDepth. Due to limited receptive fields, the first four models often struggle with accuracy in challenging regions. In contrast, our models produce more accurate and consistent depth maps by effectively capturing both local and global context. Improvements in boundary preservation and distant structure estimation are especially evident in the yellow boxes.

As visualized in Figure 4, MogaDepth delivers clearer geometric structures, sharper object boundaries, and smoother depth transitions in texture-less or distant regions—qualitative results that are consistent with the optimized SqRel and RMSE metrics in Table 2. These qualitative advantages further verify the synergistic effect of CMOGA and MambaSync modules in enhancing the structural fidelity of depth maps. To fully evaluate the model’s practical value, we further integrate the above accuracy results with complexity and inference speed analysis (detailed in the following section), as MogaDepth’s core design goal lies in balancing accuracy and efficiency for resource-constrained scenarios.

#### 4.4.2. Complexity and Speed Evaluation

We evaluated MogaDepth on both an NVIDIA RTX 3090 GPU and the Jetson Xavier edge platform, focusing on parameter count, FLOPs, and inference time—key metrics for assessing deployability in resource-constrained scenarios. As shown in Table 3, MogaDepth achieves a superior balance between model size, computational cost, and inference speed compared to the listed lightweight methods, which compensates for its lack of leading performance in partial accuracy metrics.

Specifically, under comparable input dimensions, MogaDepth-Tiny, MogaDepth-Small, and the standard MogaDepth run approximately 4%, 18%, and 13% faster than their corresponding Lite-Mono variants, respectively. This consistent speed advantage across all three model variants highlights the inherent efficiency of our Mamba-based architecture, which is a core innovation distinguishing MogaDepth from attention-based lightweight models. The key to this efficiency lies in the fundamental architectural difference between Mamba and conventional self-attention mechanisms: while Lite-Mono [10] and our model have similar parameter counts and FLOPs, attention-based models are bottlenecked by frequent memory access to large key-value matrices, especially for long-sequence high-resolution feature maps. In contrast, Mamba employs a linear recurrent formulation, which eliminates the quadratic complexity of attention. During inference, it maintains only a compact hidden state for each new token, minimizing repeated memory access and improving cache utilization. This design fundamentally reduces latency, enabling MogaDepth to achieve faster inference on both high-performance GPUs and edge devices while preserving comparable accuracy.

Combined with the KITTI accuracy results, these findings demonstrate that MogaDepth strikes a favorable trade-off between depth estimation accuracy and inference efficiency that is tailored for resource-constrained scenarios. For real-world applications such as mobile robotics and embedded autonomous driving—where computational resources are limited and real-time performance is critical—MogaDepth’s balanced design offers greater practical value than models that pursue top accuracy at the cost of efficiency.

#### 4.4.3. Make3D Results

**Figure 5:**
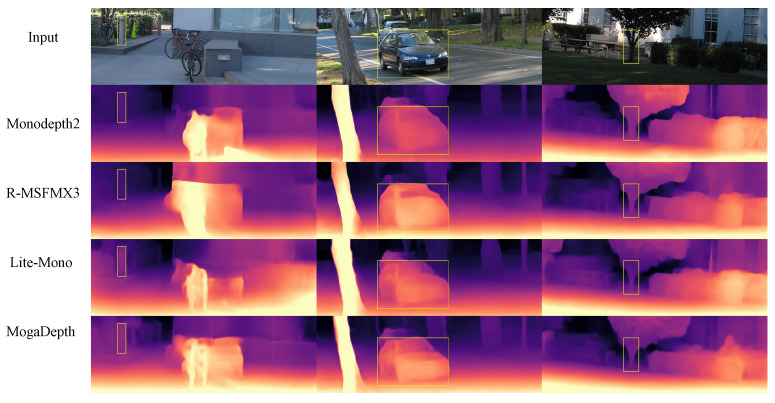
Qualitative results on the Make3D dataset. MogaDepth is compared to Monodepth2 [6], R-MSFMX3 [16] and Lite-Mono [10]. MogaDepth can perceive different sizes of objects.

As presented in Table 4 and qualitatively validated in Figure 5, the effectiveness of MogaDepth on the Make3D dataset is evidenced by consistent improvements across all evaluation metrics compared with existing methods. In particular, when compared with R-MSFMX6-GC [16], MogaDepth achieves a 10.9% reduction in Abs Rel (0.285 vs. 0.290), an 11.6% reduction in Sq Rel (2.789 vs. 2.911), a slight improvement in RMSE log (0.150 vs. 0.151), and a marginally lower RMSE (6.409 vs. 6.418).

These results suggest that MogaDepth can be effectively adapted to diverse depth distributions and remains robust in challenging scenarios, including low-texture regions and significant domain shifts. Overall, the consistent gains in both in-domain and cross-domain evaluations demonstrate that MogaDepth generalizes well beyond its training distribution and exhibits strong adaptability to unseen environments.

### 4.5. Ablation Study

#### 4.5.1. Model Architectures

To rigorously verify the design superiority of the core components (CMOGA and MambaSync), we conduct an ablation study on the KITTI benchmark under a strict fixed parameter budget constraint (3.4 M, consistent with the full MogaDepth model). The input size is fixed at 640×192 for all experiments.

Specifically, when removing a target module (MambaSync or CMOGA), we compensate for the reduced parameters by adjusting the number of convolution layers in redundant layers of the backbone.This design ensures that the performance difference between models is solely caused by the structural advantages of the proposed modules, rather than parameter scaling. All training hyper-parameters (learning rate, optimizer, epochs, etc.) are kept identical to eliminate additional interference.

Quantitative results in Table 5 demonstrate that removing either CMOGA or MambaSync leads to consistent degradation in depth estimation accuracy (e.g., increased Abs Rel and RMSE, decreased δ<1.25), even with the same total parameters. This fully validates the indispensability and rationality of our module design for balancing accuracy and efficiency.

**CMOGA blocks.** The CMOGA module enhances multi-scale and mid-order feature integration, which is often underexplored in lightweight monocular depth estimation. Ablation results confirm that CMOGA significantly improves the network’s ability to capture both fine-grained geometric details and global scene structure.

**MambaSync blocks.** MambaSync balances global context modeling and local feature preservation using hierarchical state-space modeling and lightweight convolutions. Ablation demonstrates that it effectively improves depth prediction accuracy while maintaining computational efficiency, validating its importance for real-time, lightweight monocular depth estimation.

#### 4.5.2. Dilation Rates

We study the impact of different dilation rate settings in the CMOGA module for lightweight monocular depth estimation. Three configurations are evaluated:

- **Default (ours):** For Stage 1 and Stage 2, SA blocks employ (1,2,3) repeated *n* times, followed by a final block of (1,2,5). For Stage 3, the CMOGA sequence is repeated three times, each consisting of (1,2,3) repeated 2 times followed by (1,2,5). In other words, the full sequence is 3×[(1,2,3)×n+(1,2,5)].
- **Alternative 1:** Follows the same stage-wise structure as Default, but replaces all occurrences of (1,2,3) with (1,2,1) and (1,2,5) with (1,2,3) in the sequences.
- **Alternative 2:** Follows the same stage-wise structure as Default, but replaces all occurrences of (1,2,3) with (1,2,1).
- **MogaNet baseline:** Original MogaNet setup: (1,2,1) repeated *n* times, with the last block (1,2,3) in each stage.

As shown in Table 6 our default configuration effectively enhances mid-order feature fusion while preserving multi-scale context, making it particularly suitable for lightweight monocular depth estimation. Alternative configurations show minor reductions in performance, supporting the choice of our hyperparameters as both effective and reasonable for the task.

#### 4.5.3. Module Comparison: CDC vs. CMOGA

To further validate the superiority of our CMOGA in capturing mid-order features over existing progressive dilation designs, we conduct a controlled experiment where we replace the CMOGA module in our full model with a CDC module while keeping all other components (including LGFI) and architectural settings identical. This ensures a fair comparison that isolates the impact of the core aggregation module design. Quantitative results in Table 7 show that our CMOGA-based model consistently outperforms the CDC-based counterpart across all major metrics on the KITTI benchmark. Notably, the improvement in RMSE (from 4.589 to 4.504) and $\delta_{1}$ (from 0.886 to 0.891) highlights CMOGA’s enhanced capability in preserving structural details and depth accuracy. These results confirm that explicit modeling of mid-order feature interactions, as implemented in CMOGA, provides more effective feature representations than CDC’s progressive dilation approach, which primarily focuses on receptive field expansion.

#### 4.5.4. MambaSync Module Ablation Analysis

The MambaSync module fuses complementary local and global features, with an SE module balancing their contributions. To verify the necessity of each component, we built four variants by establishing different components, keeping all other experimental settings consistent for fairness.

As shown in Table 8, the full MambaSync module (integrating both local and global branches with the SE gating) achieves the best performance across all metrics. Removing either the local or global branch leads to a noticeable drop in accuracy, confirming that both types of features are complementary and necessary for robust depth estimation. Specifically, the absence of the local branch results in the largest degradation in Sq Rel (from 0.728 to 0.797), indicating that fine-grained geometric details are crucial for reconstructing dense depth maps. Meanwhile, removing the global branch moderately increases RMSE (from 4.504 to 4.635), underscoring the importance of long-range contextual information for overall scene coherence. Furthermore, ablating the SE gating mechanism (Local + Global without SE) causes a consistent, though slight, performance decline, which verifies that adaptive feature recalibration helps to optimally fuse the two branches. These ablation results collectively validate the design rationale of the MambaSync module and highlight the contribution of each component to the final depth estimation performance.

## 5. Conclusions

In this paper, we have presented **MogaDepth**, a lightweight and efficient architecture for self-supervised monocular depth estimation. By integrating convolutional backbones with Mamba-based components, and introducing the **CMOGA** and **MambaSync** modules, MogaDepth effectively captures mid-order feature interactions and long-range global dependencies. Extensive experiments on KITTI and Make3D demonstrate that MogaDepth achieves highly competitive performance while maintaining a compact model size and strong generalization to unseen domains. Importantly, MogaDepth also offers significant improvements in inference speed on edge devices, achieving up to 13% faster processing without sacrificing accuracy, highlighting its suitability for real-time applications in resource-constrained environments. Ablation studies further validate the contribution of both CMOGA and MambaSync to improved depth accuracy and feature representation.

Future work will focus on further enhancing mid-order feature modeling, integrating multi-modal information, and optimizing performance for resource-constrained platforms, with the goal of improving depth estimation under challenging scenarios such as extreme lighting conditions and dynamic environments. 

## Figures and Tables

**Table 1:** Three variants of the proposed depth encoder. Each CMOGA stage adopts a sequence of dilation rates for initial blocks and a distinct combination for the final block, as detailed below.

Output Size	Layers	MogaDepth-Tiny	MogaDepth-Small	MogaDepth
640×192	Input			
320 × 96	Conv Stem	3×3,32, stride=2	3×3,48, stride=2	3×3,48, stride=2
		[3×3,32]×2	[3×3,48]×2	[3×3,48]×2
160×48	Downsampling	3×3,32, stride=2	3×3,48, stride=2	3×3,48, stride=2
Stage 1	CMOGA blocks	(1,2,3)×2 + (1,2,5)	(1,2,3)×2 + (1,2,5)	(1,2,3)×2 + (1,2,5)
	MambaSync block			
80×24	Downsampling	3×3,64, stride=2	3×3,80, stride=2	3×3,80, stride=2
Stage 2	CMOGA blocks	(1,2,3)×2 + (1,2,5)	(1,2,3)×2 + (1,2,5)	(1,2,3)×2 + (1,2,5)
	MambaSync block			
40×12	Downsampling	3×3,128, stride=2	3×3,128, stride=2	3×3,128, stride=2
Stage 3	CMOGA blocks	2×[(1,2,3)×2+(1,2,5)]	2×[(1,2,3)×2+(1,2,5)]	3×[(1,2,3)×2+(1,2,5)]
	MambaSync block			
#Params (M)		2.4	2.8	3.4

Note: Each CMOGA stage repeats the sequence “(1,2,3)×2+(1,2,5)” multiple times. Here, the “+(1,2,5)” indicates the last CMOGA block of each repeated unit, not the final block of the entire stage.

**Table 2:** Comparison of MogaDepth with several recent representative methods on the KITTI benchmark under the Eigen split [39]. Unless otherwise specified, all input images are resized to 640×192. The best and second-best results are indicated in **bold** and underlined, respectively. “M” denotes training on KITTI monocular video sequences; “M+Se” indicates the use of monocular videos combined with semantic segmentation supervision; “M*”: input resolution of 1024×320; “M†”: models trained without ImageNet pretraining [38]. Detailed architectural differences between MogaDepth-tiny, MogaDepth-small, and the standard MogaDepth are documented in Table 1.

Method	Year	Data	Depth Error (↓)				Depth Accuracy (↑)			Model Size (↓)
			Abs Rel	Sq Rel	RMSE	RMSE log	δ<1.25	$\delta <1.25^{2}$	$\delta <1.25^{3}$	Params.
SGDepth [21]	2020	M+Se	0.113	0.835	4.693	0.191	0.879	0.961	0.981	16.3M
HR-Depth [40]	2021	M	0.109	0.792	4.632	0.185	0.884	0.962	0.983	14.7M
MonoFormer [33]	2022	M	0.108	0.806	4.594	0.184	0.884	0.963	0.983	23.9M
Lite-Mono-tiny [11]	2023	M	0.110	0.837	4.710	0.187	0.880	0.960	0.982	2.2M
Lite-Mono-small [10]	2023	M	0.110	0.802	4.671	0.186	0.879	0.961	0.982	2.5M
Lite-Mono [10]	2023	M	0.107	0.765	4.561	0.183	0.886	0.963	0.983	3.1M
Lite-Mono-8M [10]	2023	M	0.101	0.729	4.454	0.178	**0.897**	0.965	0.983	3.1M
R-MSFMX3 [16]	2024	M	0.111	0.775	4.666	0.190	0.879	0.960	0.981	3.5M
R-MSFMX3-GC [16]	2024	M	0.112	0.789	4.621	0.189	0.880	0.960	0.982	3.5M
R-MSFMX6 [16]	2024	M	0.111	0.789	4.626	0.189	0.883	0.961	0.981	3.5M
R-MSFMX6-GC [16]	2024	M	0.112	0.789	4.597	0.189	0.881	0.961	0.981	3.8M
LDA-Mono-S [41]	2024	M	0.110	0.833	4.659	0.185	0.879	0.961	0.983	**0.7M**
LDA-Mono-M [41]	2024	M	0.108	0.802	4.633	0.183	0.893	0.964	0.983	1.1M
LDA-Mono-L [41]	2024	M	0.104	0.765	4.535	0.180	0.893	0.964	0.983	2.0M
DNA-Depth-B1 [42]	2024	M	0.102	0.757	4.493	0.178	0.896	0.965	**0.984**	6.7M
ADDepth [43]	2025	M	0.106	0.712	4.425	0.180	0.889	0.965	**0.984**	6.3M
Sun et al. [44]	2025	M	**0.100**	**0.702**	**4.403**	**0.177**	0.894	**0.966**	**0.984**	10.9M
**MogaDepth-tiny(ours)**	2025	M	0.109	0.828	4.676	0.185	0.886	0.962	0.982	2.4M
**MogaDepth-small(ours)**	2025	M	0.108	0.787	4.656	0.184	0.886	0.962	0.982	2.7M
**MogaDepth(ours)**	2025	M	0.104	0.745	4.504	0.181	0.892	0.964	**0.984**	3.4M
Lite-Mono-tiny [10]	2023	M†	0.125	0.935	4.986	0.204	0.853	0.950	0.978	2.2M
Lite-Mono-small [10]	2023	M†	0.123	0.919	4.926	0.202	0.859	0.951	0.977	2.5M
Lite-Mono [10]	2023	M†	0.121	0.876	4.918	0.199	0.859	0.953	0.980	3.1M
LDA-Mono-S [41]	2024	M†	0.124	0.993	4.972	0.199	0.860	0.953	0.979	**0.7M**
LDA-Mono-M [41]	2024	M†	0.117	0.927	4.869	0.195	0.872	0.957	0.980	1.1M
LDA-Mono-L [41]	2024	M†	**0.115**	0.870	**4.730**	**0.192**	**0.876**	**0.959**	**0.981**	2.0M
**MogaDepth-tiny(ours)**	2025	M†	0.124	0.924	4.979	0.203	0.856	0.952	0.977	2.4M
**MogaDepth-small(ours)**	2025	M†	0.120	0.900	4.976	0.210	0.857	0.953	0.979	2.7M
**MogaDepth(ours)**	2025	M†	0.119	**0.866**	4.836	0.198	0.860	0.956	**0.981**	3.4M
Lite-Mono-tiny [10]	2023	M*	0.104	0.764	4.487	0.180	0.892	0.964	0.983	**2.2M**
Lite-Mono-small [10]	2023	M*	0.103	0.757	4.449	0.180	0.894	0.964	0.983	2.5M
Lite-Mono [10]	2023	M*	0.102	0.746	4.444	0.179	0.896	0.965	0.983	3.1M
Lite-Mono-8M [10]	2023	M*	**0.097**	0.710	**4.309**	**0.174**	**0.905**	0.967	**0.984**	8.7M
R-MSFMX3-GC [16]	2024	M*	0.107	0.789	4.621	0.185	0.886	0.962	0.982	5.0M
R-MSFMX6-GC [16]	2024	M*	0.103	0.693	4.363	0.180	0.894	0.965	0.983	5.3M
DNA-Depth-B1 [42]	2024	M*	**0.097**	**0.682**	4.357	**0.174**	0.902	**0.968**	**0.984**	6.7M
**MogaDepth-tiny(ours)**	2025	M*	0.104	0.752	4.460	0.180	0.895	0.964	0.983	2.4M
**MogaDepth-small(ours)**	2025	M*	0.102	0.754	4.428	0.177	**0.905**	0.965	**0.984**	2.7M
**MogaDepth(ours)**	2025	M*	0.098	0.730	4.341	**0.174**	0.904	0.967	**0.984**	3.4M

**Table 3:** Model complexity and speed evaluation. We compare parameters, FLOPs, and inference speed. The input size is 640×192, and the batch size is 16. The best and second-best results are indicated in bold and underlined.

	Full Model		Speed (ms)	
Method	Params. (M)	FLOPs (G)	RTX 3090	Jetson Xavier
R-MSFMX3 [16]	5.0	19.8	4.7	22.3
R-MSFMX6 [16]	5.3	34.5	7.1	41.7
Lite-Mono-tiny [10]	2.2	2.9	1.8	12.7
Lite-Mono-small [10]	2.5	4.8	2.2	19.2
Lite-Mono [10]	3.1	5.1	2.3	20.0
Lite-Mono-8m [10]	8.7	11.2	3.4	32.2
**MogaDepth-tiny(ours)**	2.4	2.9	**1.4**	**12.2**
**MogaDepth-small(ours)**	2.7	4.8	1.8	15.7
**MogaDepth(ours)**	3.4	5.1	2.0	17.4

**Table 4:** Comparison of the proposed MogaDepth to some other methods on the Make3D [19] dataset. All models are trained on KITTI [18] with an image resolution of 640×192. The best and second-best results are indicated in bold and underlined.

Method	Abs Rel	Sq Rel	RMSE	RMSE Log
DDVO [45]	0.387	4.720	8.090	0.204
Monodepth2 [6]	0.322	3.589	7.417	0.163
R-MSFMX6-GC [16]	0.290	2.911	6.418	0.151
Lite-Mono [10]	0.305	3.060	6.981	0.158
DNA-Depth-B0 [42]	0.301	2.845	6.833	0.156
DNA-Depth-B1 [42]	0.310	3.026	6.862	0.158
ADD-Depth [43]	0.305	3.018	6.855	0.155
**MogaDepth(ours)**	**0.285**	**2.789**	**6.409**	**0.150**

**Table 5:** Ablation Study on Core Modules of MogaDepth (Fixed Parameter Budget). All models are trained and tested on the KITTI dataset with input size 640×192 and fixed total parameters of 3.4 M. The best result is indicated in bold.

Architecture Variant	Module Config.	Speed (ms)	Abs Rel	Sq Rel	RMSE	RMSE log	δ<1.25	$\delta <1.25^{2}$	$\delta <1.25^{3}$
MogaDepth (Full Model)	CMOGA + MambaSync	2.0	**0.104**	**0.728**	**4.504**	**0.181**	**0.891**	**0.964**	**0.983**
w/o MambaSync (Compensated)	CMOGA only	1.8	0.108	0.862	4.785	0.186	0.884	0.960	0.981
w/o CMOGA (Compensated)	MambaSync only	1.3	0.118	0.905	4.912	0.190	0.877	0.957	0.979

Note: Parameter compensation is implemented by adding convolution layers to redundant layers to maintain consistent parameter budget.

**Table 6:** Ablation study on different dilation rates. The best result is indicated in bold.

NO.	Abs Rel	Sq Rel	RMSE	RMSE log	$\delta_{1}$	$\delta_{2}$	$\delta_{3}$
1	**0.104**	**0.728**	**4.504**	**0.181**	**0.892**	**0.964**	**0.983 **
2	0.105	0.788	4.602	0.183	0.890	0.962	0.983
3	0.104	0.798	4.622	0.183	0.891	0.962	0.983
4	0.105	0.765	4.546	0.182	0.889	0.963	0.982

**Table 7:** Module comparison: CDC vs. CMOGA. Both models share identical architecture except for the core aggregation module, ensuring a fair evaluation of module effectiveness. All models are trained and tested on KITTI with input size 640×192. The best result is indicated in bold.

Architecture	Params.	Speed (ms)	Abs Rel	Sq Rel	RMSE	RMSE log	δ<1.25	$\delta <1.25^{2}$	$\delta <1.25^{3}$
**CMOGA-based (full model)**	3.408M	2.0	**0.104**	**0.728**	**4.504**	**0.181**	**0.891**	**0.964**	**0.983**
**CDC-based**	3.395M	1.9	0.107	0.781	4589	0.182	0.886	0.963	0.983

**Table 8:** Ablation study on MambaSync module. The best result is indicated in bold.

Structure	Abs Rel	Sq Rel	RMSE	RMSE Log	$\delta_{1}$	$\delta_{2}$	$\delta_{3}$
local branch + gobal branch + SE	**0.104**	**0.728**	**4.504**	**0.181**	**0.892**	**0.964**	**0.983**
local branch + SE	0.108	0.797	4.762	0.185	0.886	0.962	0.981
global branch + SE	0.106	0.768	4.635	0.184	0.888	0.962	0.982
local branch + gobal branch	0.106	0.764	4.622	0.183	0.887	0.963	0.983

## Data Availability

The data used to support the findings of this study are publicly available and were obtained from established repositories. The KITTI dataset is accessible at http://www.cvlibs.net/datasets/kitti/ accessed on 15 March 2025, and the Make3D dataset can be retrieved from http://make3d.cs.cornell.edu/data.html accessed on 12 April 2025. No new original datasets were generated during the conduct of this study.
